# Sympathetic and Parasympathetic Modulation of Pupillary Unrest

**DOI:** 10.3389/fnins.2020.00178

**Published:** 2020-03-11

**Authors:** Andy Schumann, Stephanie Kietzer, Juliane Ebel, Karl Jürgen Bär

**Affiliations:** ^1^Lab for Autonomic Neuroscience, Imaging and Cognition (LANIC), Department of Psychosomatic Medicine and Psychotherapy, Jena University Hospital, Jena, Germany; ^2^Department of Psychiatry and Psychotherapy, Jena University Hospital, Jena, Germany

**Keywords:** pupillometry, heart rate variability, blood pressure, skin conductance, respiration

## Abstract

Pupillary unrest is an established indicator of drowsiness or sleepiness. How sympathetic and parasympathetic activity contribute to pupillary unrest is not entirely unclear. In this study, we investigated 83 young healthy volunteers to assess the relationship of pupillary unrest to other markers of the autonomic nervous system. Sample entropy (SE) and the established pupillary unrest index (PUI) were calculated to characterize pupil size variability. Autonomic indices were derived from heart rate, blood pressure, respiration, and skin conductance. Additionally, we assessed individual levels of calmness, vigilance, and mood. In an independent sample of 26 healthy participants, we stimulated the cardiovagal system by a deep breathing test. PUI was related to parasympathetic cardiac indices and sleepiness. A linear combination of vagal heart rate variability [root mean square of heart beat interval differences (RMSSD)] and skin conductance fluctuations (SCFs) was suited best to explain interindividual variance of PUI. Complexity of pupil diameter (PD) variations correlated to indices of sympathetic skin conductance. Furthermore, we found that spontaneous fluctuations of skin conductance are accompanied by increases of pupil size. In an independent sample, we were able to corroborate the relation of PUI with RMSSD and skin conductance. A slow breathing test enhanced RMSSD and PUI proportionally to each other, while complexity of PD dynamics decreased. Our data suggest that the slow PD oscillations (*f* < 0.15 Hz) quantified by PUI are related to the parasympathetic modulation. Sympathetic arousal as detected by SCFs is associated to transient pupil size increases that increase non-linear pupillary dynamics.

## Introduction

The pupil seems to offer unique insights into the human mind. For centuries, the pupillary system has been in the focus of psychophysiological research (Sirois and Brisson, 2014). Charles Darwin discovered pupillary reactions to fear and other emotions in animals (Darwin, 1872). Hess and colleagues reported that human pupils dilate in response to appealing images and statements (Hess and Polt, 1960; Hess, 1965; Hess et al., 1965). Since then, pupillometric investigations have been used in numerous studies to investigate emotional responses to visual (Bradley et al., 2008) or auditory presentation (Partala and Surakka, 2003; Gingras et al., 2015). Dilation of the pupils can also indicate cognitive load (Steinhauer et al., 2004; Eckstein et al., 2017). Pupil size has been shown to react to various types of cognitive demands, like memory load (Beatty, 1982), arithmetic tests (Võ et al., 2008), or speech processing (Stanners et al., 1979; Zekveld et al., 2010).

Moreover, the pupils are of great importance in clinical routine to evaluate autonomic function. The pupil’s reaction to light is part of every clinical examination (Cross, 1993). In contrary, little is known about pupillary fluctuations at rest. Lowenstein and Loewenfeld (1952) described spontaneous pupil diameter (PD) oscillations with high amplitude associated with sleepiness, referred to as “fatigue waves” (Lowenstein et al., 1963). The characteristic pattern of pupil size fluctuations can be quantified by the pupillary unrest index (PUI) which estimates the deviation of the PD at low frequencies (Lüdtke et al., 1998). In order to standardize the assessment of pupillary unrest, the pupillary sleepiness test was introduced (Wilhelm et al., 1998; Eggert et al., 2012). PUI is usually recorded over 11 min in a dark and quiet environment.

Borgdorff (1975) discovered slow changes of pupil size in phase with breathing. In several publications, the respiratory influence on pupil size fluctuations was investigated (Daum and Fry, 1981; Ohtsuka et al., 1988; Yoshida et al., 1994; Calcagnini and Lino, 1997; Onorati et al., 2013). Generally, sino-aortic baroreceptors are deemed responsible mediating respiratory changes of blood pressure that can be discovered in pupil size variation (Borgdorff, 1975; Häbler et al., 1994). Calcagnini et al. (2000, 2001) confirmed this explanation by stimulation of baroreceptors.

Furthermore, non-linear fluctuations seem to contribute to pupillary unrest (Mesin et al., 2013; Onorati et al., 2015; Schumann et al., 2015; Villalobos-Castaldi et al., 2016). The variety of influencing factors make spontaneous pupil size variations at rest complicated to quantify and to interpret (Usui and Stark, 1982; Mesin et al., 2013; Schumann et al., 2017b).

Both the sympathetic and parasympathetic outflow impact on pupillary muscles and modulate the size of the pupil. How pupillary unrest is affected by sympathetic activation is still illusive. Similar to pupillary reactions, skin conductance responds to emotional stimulation and cognitive load (Kohlisch and Schaefer, 1996; Najström and Jansson, 2007; Reimer and Mehler, 2011). In contrast, the electrodermal system is innervated by sympathetic sudomotor nerves only. Skin conductance reactions recorded at the hand have a latency of about 2–2.5 s due to the slow signal transmission via unmyelinated fibers (Lim et al., 2003; Bradley et al., 2008). Without a given external stimulus, spontaneous skin conductance fluctuations (SCFs) indicate sympathetic tone in face of stress or anxiety etc. (see review by Kreibig, 2010).

Since changes of the pupillary motility have been discovered in several diseases (Bär et al., 2008; Graur and Siegle, 2013), the assessment of pupillary fluctuations promises some clinical value. Recently, we demonstrated differences of pupillary unrest in patients suffering from major depression (Schumann et al., 2017a). Heart rate, skin conductance, and PUI were shown to accurately classify patients and controls (accuracy of 88.5%). In healthy subjects, PUI correlated mainly with vagal cardiovascular measures. However, the sample was rather small and somewhat heterogeneous with respect to age and gender.

Here, we investigated a larger group of healthy volunteers. We aimed to replicate and further investigate the association of pupillary unrest with other autonomic indices. An additional measure complexity of pupil size variations was estimated to quantify non-linear pupillary dynamics. Furthermore, a deep breathing test was applied in order to modulate cardiovagal function to gain insights on the dependency of pupillary dynamics on autonomic cardiac status.

## Materials and Methods

### Study Design and Participants

We assessed physiological data at rest in 83 healthy volunteers [59 females, age: 23 ± 2 years, body mass index (BMI): 21.9 ± 2.6]. The multidimensional test for mental states (MDBF) was used to evaluate subjective ratings of mood, calmness, and vigilance (Steyer et al., 1994). Demographic data and individual ratings of the three dimensions of the mental state are depicted in Table 1.

**TABLE 1:** Demographic data of the sample including 83 healthy volunteers.

Parameter	Mean	SD	Min	Max
**Demographic data**				
Age [y]	24	4	20	32
BMI [kg/m^2^]	22.0	2.6	17.2	30.0
Male	*n* = 20			
Females	*n* = 63			
**Smoking**				
*smokers*	*n* = 23			
<5 cigarettes/d	*n* = 11			
5–10 cigarettes/d	*n* = 5			
>10 cigarettes/d	*n* = 7			
**Mental state**				
Mood	33.6	5.3	15	40
Vigilance	28.5	6.3	14	40
Calmness	31.1	5.7	18	40

*BMI: body mass index.*

Healthy subjects had no present or past history of psychiatric, neurological, or other clinically significant disorders. This was excluded by taking the medical history and by a full clinical examination of all subjects. All participants gave their informed written consent in accordance with the protocol approved by the Ethics Committee of Jena.

The examination room was quiet and fully shaded with a low intensity ambient light source. Additionally, participants wore headphones to be isolated from a potential surrounding noise. Via a monitor fixed over the couch a dark gray ellipse was displayed on light gray background as fixation anchor. Room temperature was controlled to 22°C. Resting recordings were conducted in supine position for 15 min. The first 5 min were not analyzed, to exclude the adjustment period to the environment.

In 26 subjects (see Table 2), resting recordings were followed by a deep breathing test. Subjects were instructed to breathe at a fixed rate of 6 breaths/min for 5 min (Low et al., 2013). An audio track of deep ventilatory noises was given via headphones to indicate the aspired respiration rate without any change of visual presentation.

**TABLE 2:** Demographic data of the test sample including 26 healthy volunteers.

Parameter	Mean	SD	Min	Max
**Demographic data**				
Age [y]	37	13	20	63
BMI [kg/m^2^]	24.1	2.7	19.3	32.3
Male	*n* = 15			
Females	*n* = 11			
**Smoking**				
*smokers*	*n* = 5			
<5 cigarettes/d	*n* = 2			
5–10 cigarettes/d	*n* = 1			
>10 cigarettes/d	*n* = 2			

*BMI: body mass index.*

### Data Acquisition and Preprocessing

We used the MP150 system (BIOPAC Systems Inc., Goleta, CA, United States) to record multiple physiological signals simultaneously at 1000 Hz sampling rate (Schumann et al., 2017c). ECG was acquired by three electrodes on the chest according to an adjusted Einthoven triangle. The non-invasive blood pressure was measured continuously by the vascular unloading technique. ECG and blood pressure were band-pass filtered between 0.05 and 35 Hz. Abdominal and thoracic respiratory movement were recorded by two individual strain gauge transducers and low pass filtered at 10 Hz. Skin conductance was measured by constant voltage technique at the left hand’s palm with electrodes placed on the thenar and hypothenar eminence.

Heart beats were extracted automatically and checked manually offline. Artifacts and ectopic beats were detected and interpolated using an adaptive filtering technique (Wessel et al., 2000). Maximum and minimum blood pressure values embedded in one cardiac cycle were transferred to systolic and diastolic blood pressure (DBP) time series.

The pupil size was assessed every 4 ms by the infrared camera system RED 250 (SensoMotoric Inc., Boston, MA, United States). Blinks distorting PD recordings as sudden drops with duration typically less than half a second (Schleicher et al., 2008) were eliminated using a median filter with 1000 ms time window followed by temporal smoothing (400 ms). This procedure removes blinks reliably without considerable influence on the underlying PD signal.

### Skin Conductance, Cardiovascular, and Respiratory Indices

The heart rate HR and its short-term variability root mean square of heart beat interval differences (RMSSD) were estimated according to the established standard procedures (TaskForce, 1996). Additionally, respiratory sinus arrhythmia was calculated using the peak-valley-approach. Mean values of systolic blood pressure (SBP) and DBP, and breathing rate (BR) were assessed. Variability of blood pressure and respiratory cycles were quantified using the standard deviation (sdRC, sdSBP, sdDBP). Baroreflex sensitivity was estimated by the sequence method (Wessel et al., 2000). We analyzed bradycardic baroreflex sequences, i.e., the over three consecutive increases of beat-to-beat intervals accompanied by three increasing SBP values.

Spontaneous fluctuations of skin conductance (SCF, non-specific responses) were extracted from the raw signal, exploiting their typical shape (Lim et al., 1997). Skin conductance level (SCL) was estimated by averaging the whole signal. SCL and SCF were log-transformed.

Rapid regulation of heart rate as measured by RMSSD and BRS are supposed to indicate parasympathetic cardiac function (Hayano et al., 1991; Malik et al., 1996). As skin conductance is determined by sympathetic sudomotor activity, SCL and SCF are used to quantify sympathetic arousal (Bach et al., 2010).

### Phase-Rectified Signal Averaging (PRSA)

**FIGURE 1:**
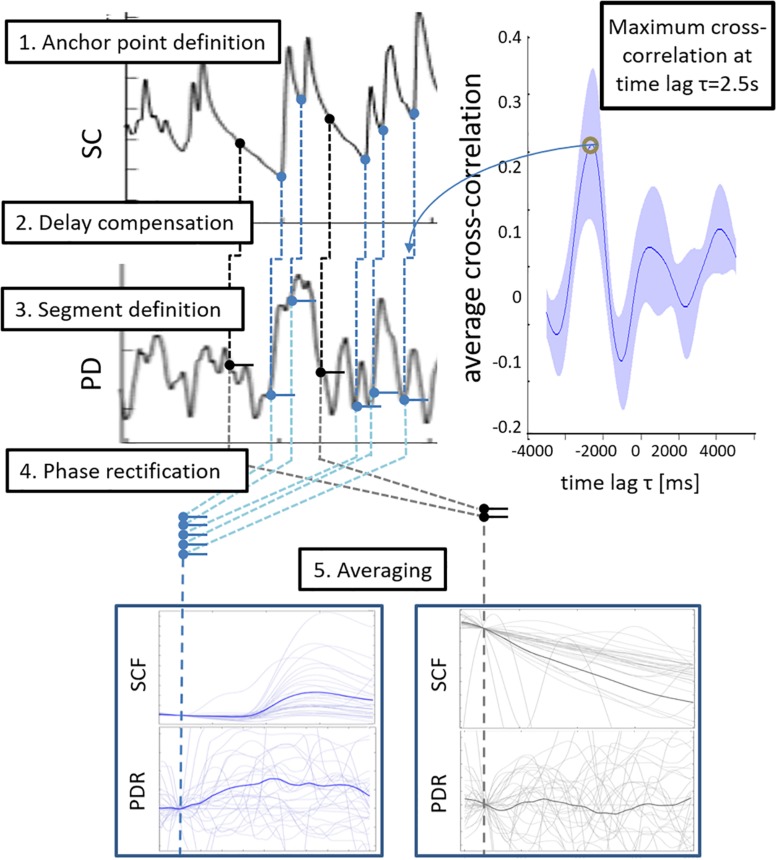
Schematic illustration of the bivariate phase rectified signal averaging (PRSA) procedure. Step 1: Onsets of skin conductance fluctuations (SCF) served as anchor points (blue circles). Randomly defined anchor points were used as reference condition (black circles). Step 2: Anchor points were transferred to the pupil diameter signal (PD) with a temporal delay of 2.5 s as the mean cross correlation function of skin conductance and PD changes reached its maximum at this time lag. Step 3: Delay-compensated anchor points were used to extract segments of pupil diameter responses (PDR). Step 4: Segments were phase-rectified and normalized. Step 5: PDR segments were averaged and analyzed.

To test whether non-specific fluctuations of skin conductance (SCF) are accompanied by pupillary responses, we extracted and averaged segments of the pupillary signal based on SCF occurrence. Phase-rectified signal averaging (PRSA) is a framework to detect and analyze recurrent patterns in non-stationary signals (Bauer et al., 2006). In the bivariate approach, a trigger signal is used to define anchor points that indicate the occurrence of a certain pattern in another signal. We used the onsets of SCF as anchors to assess the concurrent change of pupil size (blue circles in step 1, Figure 1, see Schumann et al., 2019).

We accounted for the significantly higher reaction delay of the electrodermal system (about 2–2.5 s, Lim et al., 1999; Lim et al., 2003; Rachow et al., 2011) when compared to the pupillomotor system (about 0.2 s, Korn and Bach, 2016). Therefore, the cross-correlation function of the temporal derivatives of both signals were estimated, normalized (divided by maximum), and averaged over all subjects. A representative time lag was extracted when skin conductance and pupil size changes are synchronized (maximum average cross-correlation). This time span was used to delay the anchor time points in the pupillary signal (step 2, Figure 1).

Pupil diameter responses (PDRs) over 7 s were extracted and normalized to the average pupil size in an interval 1 s prior to the anchor time point (baseline) (step 3 and 4 in Figure 1). PDRs were averaged per subject and characterized by the maximum pupil dilation and area under the curve (AUC) (step 5 in Figure 1).

To evaluate specificity of the concurrent change of pupil size, a control condition was designed by using non-SCF time points for bivariate PRSA (black circles in step 1 in Figure 1). The same number of time points as SCF actually detected in the respective subject was randomly chosen (at least 1 s distance to an SCF) as anchor points using a self-written MATLAB script based on the built-in function *randperm.m* (R2012a, The MathWorks, Natick, MA, United States). Changes of pupil size and skin conductance were extracted, averaged, and quantified the same way as before.

### Complexity of Pupillary Dynamics

Sample entropy (SE) quantifies complexity of a time series by estimating the probability that similar subseries also match at the next point of comparison. It is defined as the negative natural logarithm of a conditional probability estimate that series of length *m* match within tolerance *r* at the current and the next point (Richman and Moorman, 2000). The time series of PD sampled at 100 ms was analyzed using *m* = 5 and *r* = 0.2^∗^sd_PD,_ i.e., standard deviation of the time series.

### Pupillary Unrest Index

The PUI was introduced to evaluate sleepiness by quantifying spontaneous fluctuation (Lüdtke et al., 1998). According to the standard calculation of PUI, mean diameter values in non-overlapping segments lasting L = 640 ms were extracted. This is a procedure similar to low-pass filtering in order to exclude high frequency noise form PUI computation. Therefore, PUI primarily quantifies slow pupil size oscillations below 1.56 Hz. Absolute differences of these values were summed up and averaged per minute (Lüdtke et al., 1998).

### Regression Analysis

Bivariate linear dependencies between pupillary unrest to behavioral measures and autonomic indices were assessed by Pearson’s correlation coefficient *r* and respective *p*-value. In correlative analyses, statistical thresholds were corrected for multiple comparisons using false discovery rate (Benjamini and Hochberg, 1995). Statistical significance with 5% type-I error was assumed at *p* < 0.006. A multiple regression model was used to assess to which extent interindividual variance of PUI can be explained by other observed autonomic parameters. Correlational analyses were performed in the replication sample of 26 subjects at rest and during deep breathing. Additionally, autonomic and pupillometric indices were compared in the two conditions using a two-sample *t*-test (rest vs. deep breathing).

## Results

### Indices of Autonomic Function and Pupillary Unrest

Estimates of pupillary unrest, heart rate, blood pressure, respiration, and skin conductance are listed in Table 3. Subjects in this sample had a mean heart rate of HR = 67/min with a variability of RMSSD = 55.7 ms and relatively low blood pressure values of 109/67 mmHg (systolic/diastolic) on average. There were no significant differences between female and male participants (Table 3).

**TABLE 3:** Indices of the cardiovascular system, respiration, skin conductance, and pupil size.

Parameter	All participants [83]	Female participants [59]	Male participants [24]
**Cardiovascular indices**			
HR [min^–1^]	67 ± 7	68 ± 8	67 ± 9
RMSSD [ms]	55.7 ± 23.9	53.6 ± 24.1	57.1 ± 22.5
SBP [mmHg]	109 ± 17	107 ± 16	121.7 ± 19.2
DBP [mmHg]	67 ± 9	67 ± 8	69 ± 7
sdSBP [mmHg]	5.46 ± 7.30	5.20 ± 6.95	6.17 ± 5.02
sdDBP [mmHg]	3.90 ± 5.13	3.84 ± 4.88	3.98 ± 2.66
BRS [ms/mmHg]	26.2 ± 15.9	25.9 ± 18.4	27.9 ± 16.3
**Respiration**			
BR [min^–1^]	17 ± 3	17 ± 3	16 ± 3
sdRC [ms]	625 ± 343	580 ± 365	643 ± 595
**Skin conductance**			
SCL [μS]	6.58 ± 5.92	6.45 ± 5.75	7.44 ± 6.1
SCF [min^–1^]	4.15 ± 5.64	3.61 ± 4.35	5.87 ± 5.09
**Pupil diameter**			
Mean PD [mm]	4.17 ± 0.73	4.17 ± 0.71	4.18 ± 0.91
PUI [mm/min]	12.4 ± 4.5	12.6 ± 4.7	11.4 ± 4.6
Sample entropy	0.623 ± 0.155	0.601 ± 0.156	0.652 ± 0.157

*HR: mean heart rate, RMSSD: root mean square of heart beat interval differences, SBP: systolic blood pressure, DBP: systolic blood pressure, sdSBP: standard deviation of SBP, sdDBP: standard deviation of DBP, BRS: baroreflex sensitivity, BR: breathing rate, sdRC: standard deviation of respiratory cycles, SCL: skin conductance level, SCF: skin conductance fluctuations, Mean PD: mean pupil diameter, PUI: pupillary unrest index.*

The standard PUI and SE of PD was estimated to quantify slow variations and complexity of pupil size.

### Association of Pupillary Unrest With Autonomic Indices

Sample entropy of PD was proportional to skin conductance parameters (SCL, SCF, see Table 4). PUI correlated with vagal cardiovascular indices (RMSSD, BRS). With regard to the mental state, a significant correlation of PUI and vigilance was found (*r* = −0.354, *p* < 0.006). A linear regression model revealed that the variance of PUI can be explained by RMSSD (*b* = 0.476) and SCF (*b* = −0.269) to an extent of *R*^2^ = 0.30 (*p* < 0.001).

**TABLE 4:** Correlation analysis of pupillary dynamics and autonomic indices.

	PUI	SE
HR	–0.187	0.154
RMSSD	0.326*	–0.048
SBP	–0.059	0.125
DBP	–0.148	0.073
sdSBP	0.053	0.051
sdDBP	0.052	0.040
BRS	0.327*	–0.057
BR	–0.003	–0.114
sdRC	–0.018	0.043
SCL	–0.150	0.454*
SCF	–0.266	0.316*

HR: mean heart rate, RMSSD: root mean square of heart beat interval differences SBP: systolic blood pressure, DBP: systolic blood pressure, sdSBP: standard deviation of SBP, sdDBP: standard deviation of DBP, BRS: baroreflex sensitivity, BR: breathing rate, sdRC: standard deviation of respiratory cycles, SCL: skin conductance level, SCF: skin conductance fluctuations, PUI: pupillary unrest index, LE: Lyapunov exponent. * *p* < 0.006 (FDR).

**FIGURE 2:**
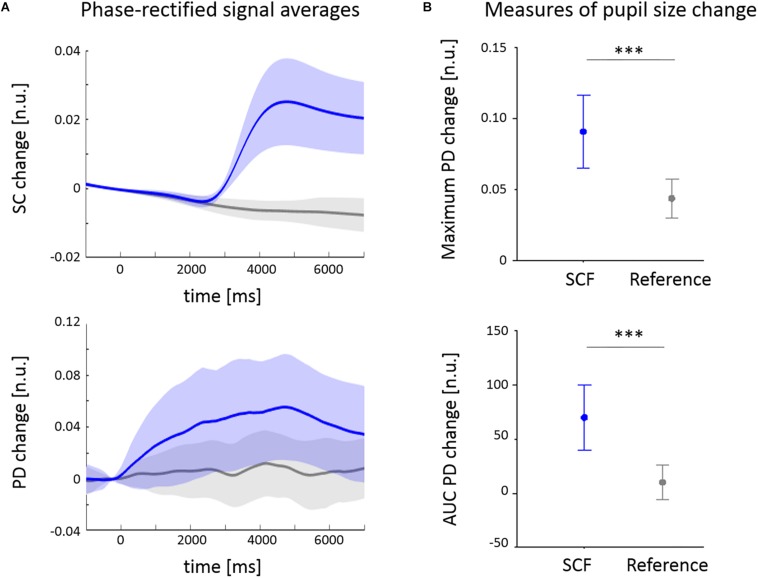
Mean changes of pupil diameter (PD) in phase with fluctuations of skin conductance (SC). **(A)** Averaged change of pupil diameter (PD) normalized to a baseline 500 ms before the reference time point *t* = 0. **(B)** Averaged fluctuations of skin conductance (SCF) used as events to analyze PD reactions. Reference time point *t* = 0 was extracted 2500 ms prior to each SCF onset. SC changes were normalized to a baseline 500 ms before the reference time point. ^∗∗∗^*p* < 0.001.

The cross-correlation function of temporal derivatives of skin conductance and pupil signals revealed a maximum correlation at a delay of 2501 ms (see Figure 1). The averaged change of PD is depicted together with the mean time course of spontaneous SCFs (SC) in Figure 2A. In gray, the segments extracted from non-SCF (randomly defined anchor points) are illustrated that served as control condition. SCFs are accompanied by PD increases of 9.1 ± 9.4% on average. The one sample *t*-test indicated a positive area under the PDR curve (70 ± 110 n.u., *p* < 0.001). Both measures were significantly higher when PDR extraction was triggered by SCF when compared to the control condition with randomly defined anchor points (both *p* < 0.001, Figure 2B). Maximum PD change was correlated to PUI (*r* = 0.545, *p* < 0.001).

### Influence of Deep Breathing

In a group of 26 subjects, a deep breathing test was performed after the resting state measurement. The test had a significant impact on autonomic indices as well as pupillary unrest (see Table 5). Respiratory measures indicated that subjects succeeded in breathing steadily at a rate of 6 breaths/min. As we expected, cardiovagal parameters such as RMSSD and BRS were enhanced during deep breathing. The test had significant effects on mean blood pressure values (SBP, DBP) but not on average heart rate. While, pupil size and PUI increased during the test, complexity of PD in terms of SE decreased.

**TABLE 5:** Influence of deep breathing test on autonomic indices and pupillary dynamics.

Parameter	Resting condition	Deep breathing	Significance
**Cardiovascular indices**			
HR [min^–1^]	64 ± 11	64 ± 10	n.s.
RMSSD [ms]	35.5 ± 17.9	56.7 ± 19.9	*p* < 0.001
SBP [mmHg]	114.1 ± 16.9	110.8 ± 18.2	*p* < 0.05
DBP [mmHg]	70.9 ± 9.2	65.3 ± 11.3	*p* < 0.001
sdSBP [mmHg]	5.31 ± 2.08	5.25 ± 1.62	n.s.
sdDBP [mmHg]	3.81 ± 2.17	3.94 ± 1.25	n.s.
BRS [ms/mmHg]	15.8 ± 10.4	24.4 ± 13.9	*p* < 0.001
**Respiration**			
BR [min^–1^]	12 ± 4	6 ± 1	*p* < 0.001
sdRC [ms]	721 ± 410	276 ± 112	*p* < 0.001
**Skin conductance**			
SCL [μS]	3.82 ± 3.4	4.18 ± 3.65	*p* < 0.05
SCF [min^–1^]	2.29 ± 2.9	5.22 ± 4.87	*p* < 0.001
**Pupil diameter**			
Mean PD [mm]	3.93 ± 0.76	4.12 ± 0.75	*p* < 0.01
PUI [mm/min]	11 ± 3.9	13.9 ± 8.7	*p* < 0.05
Sample entropy	0.647 ± 0.154	0.466 ± 0.104	*p* < 0.001

*HR: mean heart rate, RMSSD: root mean square of heart beat interval differences, SBP: systolic blood pressure, DBP: systolic blood pressure, sdSBP: standard deviation of SBP, sdDBP: standard deviation of DBP, BRS: baroreflex sensitivity, BR: breathing rate, sdRC: standard deviation of respiratory cycles, SCL: skin conductance level, SCF: skin conductance fluctuations, Mean PD: mean pupil diameter, PUI: pupillary unrest index.*

**FIGURE 3:**
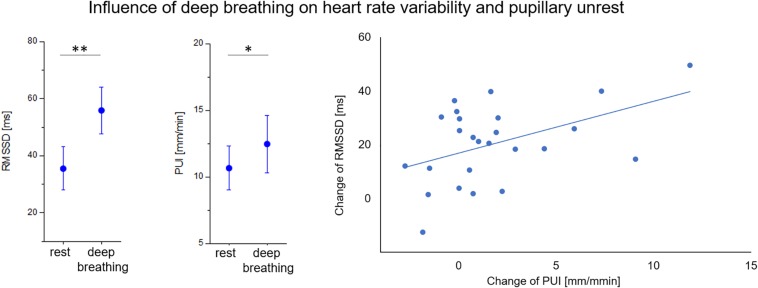
Effect of deep breathing on short-term heart rate variability (RMSSD) and pupillary unrest index (PUI). Increases of both parameters during breathing test were correlated (*r* = 0.47, *p* < 0.01). ^∗^ *p* < 0.05, ^∗∗^ *p* < 0.01.

Correlation coefficients of PD variability and autonomic indices in both conditions are depicted in Table 6. A significant correlation of PUI to RMSSD and skin conductance parameters (SCF and SCL) remained during deep breathing. Most interestingly, the increase of RMSSD during the breathing maneuver was proportional to the increase of PUI (*r* = 0.47, *p* < 0.01; see Figure 3).

**TABLE 6:** Correlation analysis of pupillary dynamics and autonomic indices at rest and deep breathing.

	Resting condition		Deep breathing	
	*PUI*	*SE*	*PUI*	*SE*
HR	0.017	0.213	0.017	0.177
RMSSD	0.462*	–0.117	0.384*	−0.439*
SBP	0.045	–0.237	0.045	0.435*
DBP	–0.138	–0.295	–0.138	0.287
sdSBP	0.028	0.223	0.028	0.414*
sdDBP	0.045	0.289	0.045	0.260
BRS	0.228	–0.343	0.228	–0.318
BR	–0.104	0.296	0.050	–0.078
sdRC	–0.116	0.070	–0.107	0.085
SCL	−0.412*	0.130	−0.569**	0.029
SCF	−0.428*	0.245	−0.422*	0.474*

HR: mean heart rate, RMSSD: root mean square of heart beat interval differences SBP: systolic blood pressure, DBP: systolic blood pressure, sdSBP: standard deviation of SBP, sdDBP: standard deviation of DBP, BRS: bradycardic baroreflex sensitivity, BR: breathing rate, sdRC: standard deviation of respiratory cycles, SCL: skin conductance level, SCF: skin conductance fluctuations, PUI: pupillary unrest index, SE: sample entropy of pupil diameter. * *p* < 0.05, ** *p* < 0.01.

## Discussion

In this study, the relation of pupillary unrest to other autonomic markers was assessed in young healthy volunteers. The classical PUI was related to parasympathetic cardiac indices and sleepiness. We found that a linear combination of vagal heart rate variability (RMSSD) and skin conductance fluctuations (SCFs) was suited best to explain interindividual variance of PUI. Complexity of PD variations correlated to indices of sympathetic skin conductance. The association of skin conductance and pupillary dynamics might be due to increases of pupil size concurrent to spontaneous SCF. In an independent sample, we were able to corroborate the correlation of pupillary unrest and heart rate variability and skin conductance. A slow breathing test enhanced cardiovagal function in this sample. While pupillary unrest was also increased, complexity of PD decreased.

The correlation of PUI with baroreflex sensitivity and heart rate variability (RMSSD) validated a link between pupillary unrest and parasympathetic cardiac modulation as reported previously (Schumann et al., 2015, 2017a). A significant correlation between PUI and individual levels of vigilance was found as well. This is in line with the idea that sleepiness induces slow rhythmic changes of pupil size that are also referred to as pupillary hippus or fatigue waves (Lowenstein and Loewenfeld, 1952, 1962; Hornung, 1968; Bouma and Baghuis, 1971). Recent research demonstrated that these pupillary oscillations are associated primarily by parasympathetic influence (Turnbull et al., 2017).

In a deep breathing test, we aimed at stimulating cardiovagal function. It has been suggested that deep breathing modulates rhythmic discharge of vagal efferent fibers without enhancing the overall level of vagal outflow (Brown et al., 1993). This leads to an increase of HRV but does not affect average HR—as we have found in our study. Since RMSSD and PUI increased proportionally to each other, we gained further evidence indicating that pupillary unrest is related to vagal modulation of cardiac function. Activity of neurons in important regulatory brainstem regions, such as the nucleus ambiguous, is modulated by respiration (see Garcia et al., 2013). At this level, systems controlling oculomotor and cardiovagal function seem to be interconnected (Borgdorff, 1975; Calcagnini et al., 2001).

While oscillatory variations of pupil size increased, non-linear complexity of pupillary dynamics decreased during deep breathing. Both during rest and breathing maneuver, SE of PD was positively correlated to skin conductance indices indicating that sympathetic arousal seems to relate to non-linear pupillary dynamics. Since an impressive co-variation of neuronal firing rate in the locus coeruleus (LC) and pupil dilation was reported (Aston-Jones and Cohen, 2005; Costa and Rudebeck, 2016), pupillary responses have been considered as a proxy of noradrenergic activity in humans (Sirois and Brisson, 2014; Eckstein et al., 2017; Schumann et al., 2018). LC activity can be decomposed into a tonic and phasic component. Tonic activity represents a kind of baseline arousal and is low during certain automatic behavior and drowsiness (Rajkowski et al., 1994; Aston-Jones et al., 1999). LC neurons become phasically activated by salient external or internal stimuli (Corbetta et al., 2008; Köhler et al., 2016). Analogously, skin conductance was divided into a tonic and phasic constituent and is most probably also influenced by the LC-noradrenergic system (Yamamoto et al., 1990). Our results indicate that phasic fluctuations of sympathetic skin conductance are synchronized with PD increases. The amplitude of pupillary responses is related to PUI, whereas the occurrence of SCF was linked to non-linear nature of PD. Using functional magnetic resonance imaging, Breeden et al. (2017) highlighted that neural correlates to fluctuations of pupil size and skin conductance have a strong overlap. In accordance to this finding, other studies suggest that changes of pupil size are mainly regulated by the salience network that contains structures related to sympathetic control (Beissner et al., 2013; Schneider et al., 2016; Breeden et al., 2017). This functional brain network is supposed to process sensory information in order to prepare executive action when salient stimuli are detected (Seeley et al., 2007). In the absence of external stimulation, thoughts or ideas during mind-wandering might elicit changes of pupil size (Smallwood et al., 2011; Grandchamp et al., 2014; Urai et al., 2017).

In a previous study, we found increased PUI values in patients with major depression that were not related to vagal HRV as in healthy controls (Schumann et al., 2017a). Furthermore, the SCF occurrence was elevated in patients. Considering the results of the present study, pupillary fluctuations associated to sympathetic arousal might have had stronger impact on PUI in patients. Most probably, internal stimuli with negative value are more predominant in patients as rumination, negative thoughts and worries are consistent symptoms related to depression (Nolen-Hoeksema et al., 2008). Therefore, elevated PUI in patients with major depression might be a consequence of a predominantly sympathetic influence on the pupil.

The reader has to keep in mind that we did not assess autonomic activity invasively. As parasympathetic and sympathetic activation are not mutually independent both branches influence each other at multiple levels of autonomic control. For instance, the activity of the sinoatrial node is modulated by parasympathetic and sympathetic impact in a widely antagonistic fashion. In contrast, skin conductance is exclusively modulated by sudomotor activity and indicates sympathetic arousal (Bach et al., 2010). Another limitation of the study is the age difference between the larger sample and the test sample that performed the deep breathing maneuver. In spite this heterogeneity, we were able to replicate a relation of pupillary unrest with heart rate variability and skin conductance.

## Conclusion

In summary, we demonstrated that pupillary unrest is related to both sympathetic and parasympathetic markers of autonomic control. Slow PD oscillations (*f* < 0.15 Hz) quantified by PUI were related to cardiovagal function. Sympathetic arousal as detected by SCFs was associated to transient pupil size increases that increase non-linear complexity of pupil size variations. The investigation of the reported relationships during different autonomic challenges might reveal further insights on the modulation of pupillary unrest.

## Data Availability Statement

The datasets generated for this study are available on request to the corresponding author.

## Ethics Statement

The studies involving human participants were reviewed and approved by the Ethikkommission der Medizinischen Fakultät der Friedrich-Schiller-Universität Jena. The patients/participants provided their written informed consent to participate in this study.

## Author Contributions

AS contributed to analysis and interpretation of the data and preparing the manuscript. SK contributed to acquisition of the data. JE contributed to quality control and preprocessing of the data. KB contributed to study conception, preparing the manuscript, and critical revision.

## Conflict of Interest

The authors declare that the research was conducted in the absence of any commercial or financial relationships that could be construed as a potential conflict of interest.
